# High school science fair: Characterizing student communication and presentation practices and their association with SEF outcomes

**DOI:** 10.1371/journal.pone.0357589

**Published:** 2026-09-11

**Authors:** Frederick Grinnell, Simon Dalley, Joan Reisch

**Affiliations:** 1 Department of Cell Biology, UT Southwestern Medical Center, Dallas, Texas, United States of America; 2 Department of Physics, Southern Methodist University, Dallas, Texas, United States of America; 3 Department of Health Data Sciences and Biostatistics, UT Southwestern O’Donnell School of Public Health, Dallas, Texas, United States of America; Auburn University, UNITED STATES OF AMERICA

## Abstract

*Obtaining, Evaluating, and Communicating Information* is one of the eight Science and Engineering Practices in the National Research Council framework that underlies Next Generation Science Standards (NGSS). Scientific inquiry requires evaluation and communication of research findings. These skills are part of scientific inquiry itself. The goal of this study was to characterize the communication practices students used during Science and Engineering Fairs (SEFs). To examine these practices, we included the following question in our online anonymous and voluntary national high school SEF surveys carried out during 2021−22 and 2022−23, “What types of communication and presentation skills did you use in your science fair project?” The possible answers were *literature review; research notebook; software to prepare tables, graphs, or images; written report; poster board preparation; PowerPoint presentation; and interview with the judges*. Literature review and research notebook are part of developing research questions and data collection. Software to prepare tables, graphs, or images, and PowerPoint presentation contribute to analyzing research and developing a presentation. Written reports, poster board preparation, and interviews with the judges provide the opportunity to present the findings. Overall, 1789 students answered the question. The percentage of students utilizing these skills ranged from 17.3% doing a literature review to 67.6% preparing a poster board. On average, students indicated the use of three skills. Poster board preparation and interview with the judges were selected by students more frequently than literature review and research notebook. Positive SEF outcomes were associated with greater use of communication and presentation skills. Among the different skills, use of a research notebook showed the strongest association. In this paper, we present the findings and discuss implications regarding use of notebooks and other communication and presentation skills in students’ SEF experiences and engagement with scientific inquiry.

## Introduction

Next Generation Science Standards (NGSS) emphasizes student experience of the practices of science as one of three essential dimensions of science education -- *students cannot comprehend scientific practices, nor fully appreciate the nature of scientific knowledge itself, without directly experiencing those practices for themselves* [1,2]. How best to accomplish hands-on practice of science and engineering remains a work in progress [3–5]. Science and engineering fairs (SEFs) provide one important opportunity for hands-on practice. Begun almost one hundred years ago, the stated goals were the same as contemporary STEM education -- *to aid in the development of the scientific leaders of the next generation and at the same time foster a better understanding of science among its laymen* [6]. Now, SEFs have become part of science education not only in the United States, but also worldwide [7–16]. At the highest level, approximately 170,000 students from 400 science fairs compete to participate in the International Science and Engineering Fair (ISEF) [17].

SEFs potentially can promote three important and desirable STEM outcomes: (i) mastery of science and engineering (S&E) practices; (ii) interest in STEM; and (iii) interest in STEM careers [7,11,18,19]. The idea that SEF participation could have a positive impact on high school students is consistent with research showing that science project-based learning advances students’ STEM understanding and interests at both the high school [20–25] and undergraduate levels [26–29]. Also, innovative high school programs that combine student participation in SEFs with student and teacher support promote STEM engagement and learning for all students including those from under-represented ethnic minorities and low socioeconomic backgrounds [30–35]. In general, student engagement in STEM research projects including SEFs fits into the self-determination model in education [], which focuses on the importance of student autonomy, competence, and community engagement [19,36–40].

The goal of our previous research has been to describe and characterize students’ experiences in high school SEFs. To accomplish this goal we developed a mixed methods approach using anonymous and voluntary surveys that combined quantitative answers with open-ended text responses. We compared student responses based on gender, ethnicity, and type of school [41,42]; learned student views about whether SEFs should be competitive vs. non-competitive and optional vs required [43,44]; and identified experiences and types of help that correlated with increased student interest in science and engineering [37,45]. We believe that having this information helps educators develop best practices leading to more effective, inclusive, and equitable SEF learning opportunities, thereby enhancing successful student participation and outcomes.

Little attention has been given to the communication practices students employ during SEF participation even though students engage in these skills as part of authentic scientific activity, and *Obtaining, Evaluating, and Communicating Information* is one of the eight Science and Engineering Practices in the National Research Council framework [1,2]. The goal of the current study was to provide a foundational analysis of the types of communication and presentation skills used by students in their SEF projects and the association of these skills with SEF outcomes. To accomplish this goal, we included the following question in our national surveys carried out during 2021−22 and 2022−23, “What types of communication and presentation skills did you use in your science fair project?” The possible answers were *literature review; research notebook; software to prepare tables, graphs, or images; written report; poster board preparation; PowerPoint presentation; and interview with the judges*. Poster board preparation and interview with the judges are skills frequently included explicitly as part of SEF scoring criteria [46]. The other skills about which we asked are elements of scientific inquiry from developing a research question to carrying out the experiments and collecting data to preparing the results for presentation to others. 1789 students answered the question. In this paper, we report and discuss the implications of their answers.

## Materials and methods

This study was approved by the UT Southwestern Medical Center (UTSW) (IRB #STU 072014−076) in August 2014 with annual continuation approvals the most recent in February, 2026. (S1 File). The study design entailed administering to students an online survey using the REDCap electronic survey and data management tool [47]. Access to the REDCap survey and data management tool at UTSW is made possible through the Department of Information Resources and Clinical and Translational Science Training Program.

Survey recipients were U.S. high school students using the Scienteer platform (www.scienteer.com) for online SEF registration, parental consent, and project management during the 2021/22 and 2022/23 school years. The Scienteer platform was funded by state and regional science fairs rather than by participatory schools. Survey enrollment was voluntary and anonymous and took place from November 18, 2021 to August 24, 2023. Although we treat the Scienteer SEF population as a national group of U.S. high school students, it should be recognized that these students come from seven U.S. states: Alabama, Louisiana, Maine, Missouri, Texas, Vermont, and Virginia. We have no information about the different locations where SEF fairs are held within each of the seven states.

Because the survey participants were minors, informed consent was obtained in two steps. In the first step, after giving consent for their student to participate in SEF, the parents decided based on the informed consent information provided by the Scienteer website if the student would have access to the survey. Second, the student decided after reading the informed consent information in the first paragraph of the survey whether to participate. Both choices were voluntary. Neither parents nor students provided identifying information in the surveys; their identities were anonymous. To prevent any misunderstanding by parents or students about the possible impact of agreeing to participate in the survey, access to the surveys was not available to students until after they finished all their SEF competitions. Scienteer did not send reminder emails, and no incentives were offered for remembering to sign back in and complete the survey.

Since 2016, when we began surveying the national Scienteer cohort of SEF students, more than 4,000 students have completed surveys, an overall response rate of about 3%. Given that student participation in the surveys involved an indirect, single electronic invitation without incentive or follow-up, this level of response was not unexpected [48–50]. Nevertheless, one limitation of our findings is that the survey respondents may not fully represent the broader population of Scienteer SEF participants. As in our previous work, we analyzed survey results for two consecutive years to increase consistency, reliability, and reproducibility. Our analyses are descriptive and exploratory in nature, intended to identify possible associations rather than demonstrate causal relationships.

The survey used for the current study can be found in supporting information (S2 File). The current version is similar to the original survey first adopted in 2015 [51]. However, since then new questions have been added about level of SEF competition; interest in a career in S&E; student ethnicity; school location (urban, suburban, rural); and reasons why or why not science fair experience increased the students’ interest in S&E. Beginning in 2021 we added the question *What types of communication and presentation skills did you use in your science fair project?* The possible answers were *literature review*; *research notebook*; *software to prepare tables, graphs, or images; written report*; *poster board preparation*; *PowerPoint presentation*; and *interview with the judges*. Students could select multiple skills independently. The students’ responses to this question are the focus of the current study and can be found in supporting information (S3 File). Given that students who participated in the survey were from seven different states and that participation was anonymous, the S3 File contains no potentially identifying information.

In the figures, survey data are summarized as frequency counts and percentages. Associations between categorical variables were evaluated using Pearson’s chi-square tests of independence (*P* values calculated without Yates correction). No adjustments were made for multiple comparisons. Wilson 95% confidence intervals were calculated for the proportions shown in Figs 2, 5, and 7.

## Results

### Overview of survey responses about communication and presentation skills

In our surveys, the final two questions ask students about the outcomes of their SEF participation. Approximately 85% of the students who began surveys answered one or both of those questions. These surveys were considered to be “complete” and used for subsequent analyses. With the completed surveys, not every student answered every other question. The figure legends specify how many students did not answer a particular question.

Throughout the manuscript we refer to skills because that is how the survey questions were framed. However, it should be emphasized that the findings represent students’ self-reported experiences rather than direct evidence of proficiency in performing the skills.

**Fig 1:**
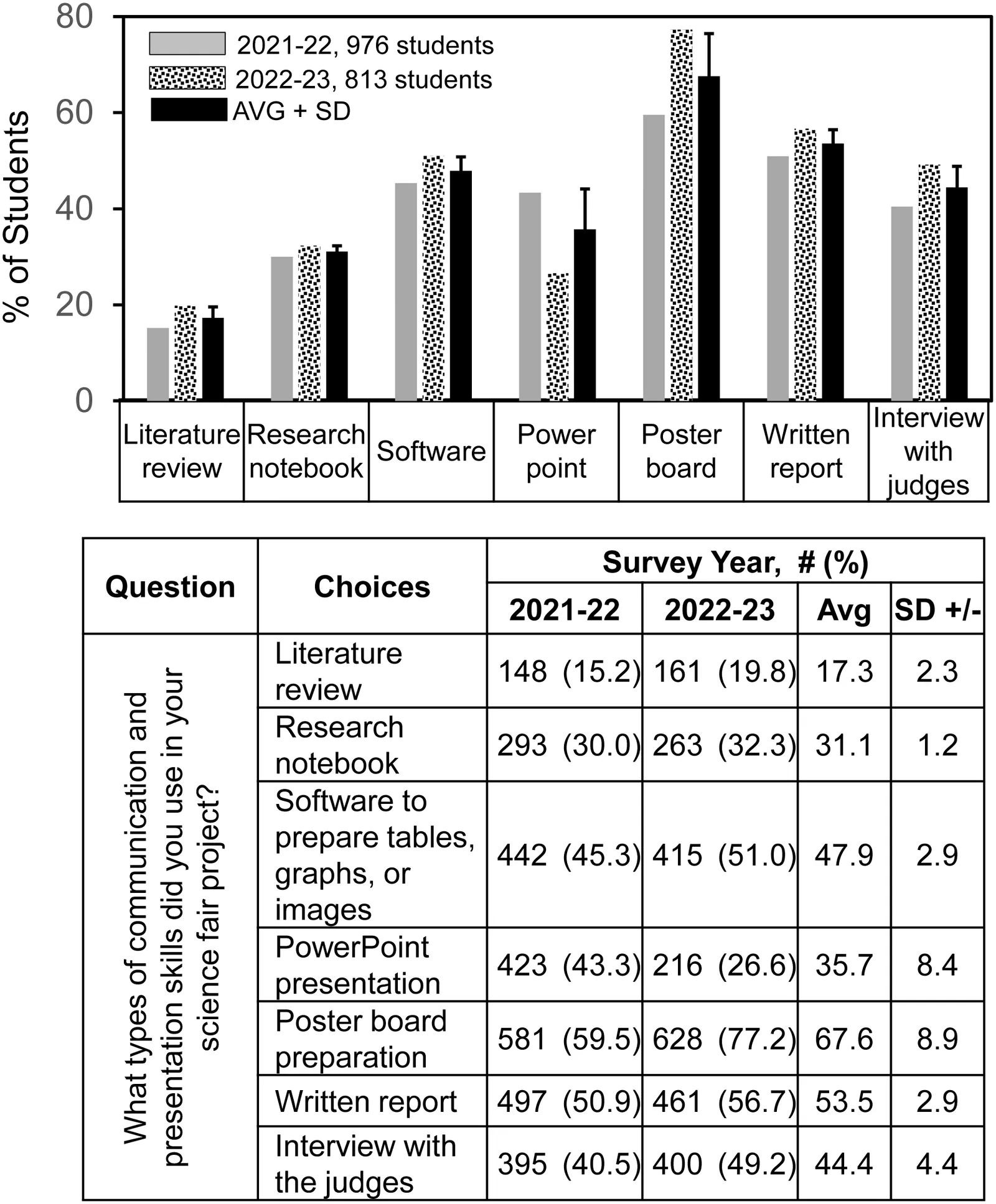
Year-to-year comparison and the averages of students’ responses to the question about communication and presentation skills used in their SEF projects. Error bars on the mean values indicate the standard deviation of the percentages observed in the two survey years.

Fig 1 shows the year-to-year comparison and the averages of students’ responses to the question about communication and presentation skills used in their SEF projects. Literature review was reported by the fewest students (15–20% across survey years). Poster board preparation was utilized by the greatest number (60–77%). The students’ responses were similar overall across the two survey years except that PowerPoint presentation and poster board showed greater variation than the other skills measured. In subsequent figures, we show the combined results for the two survey years.

**Fig 2:**
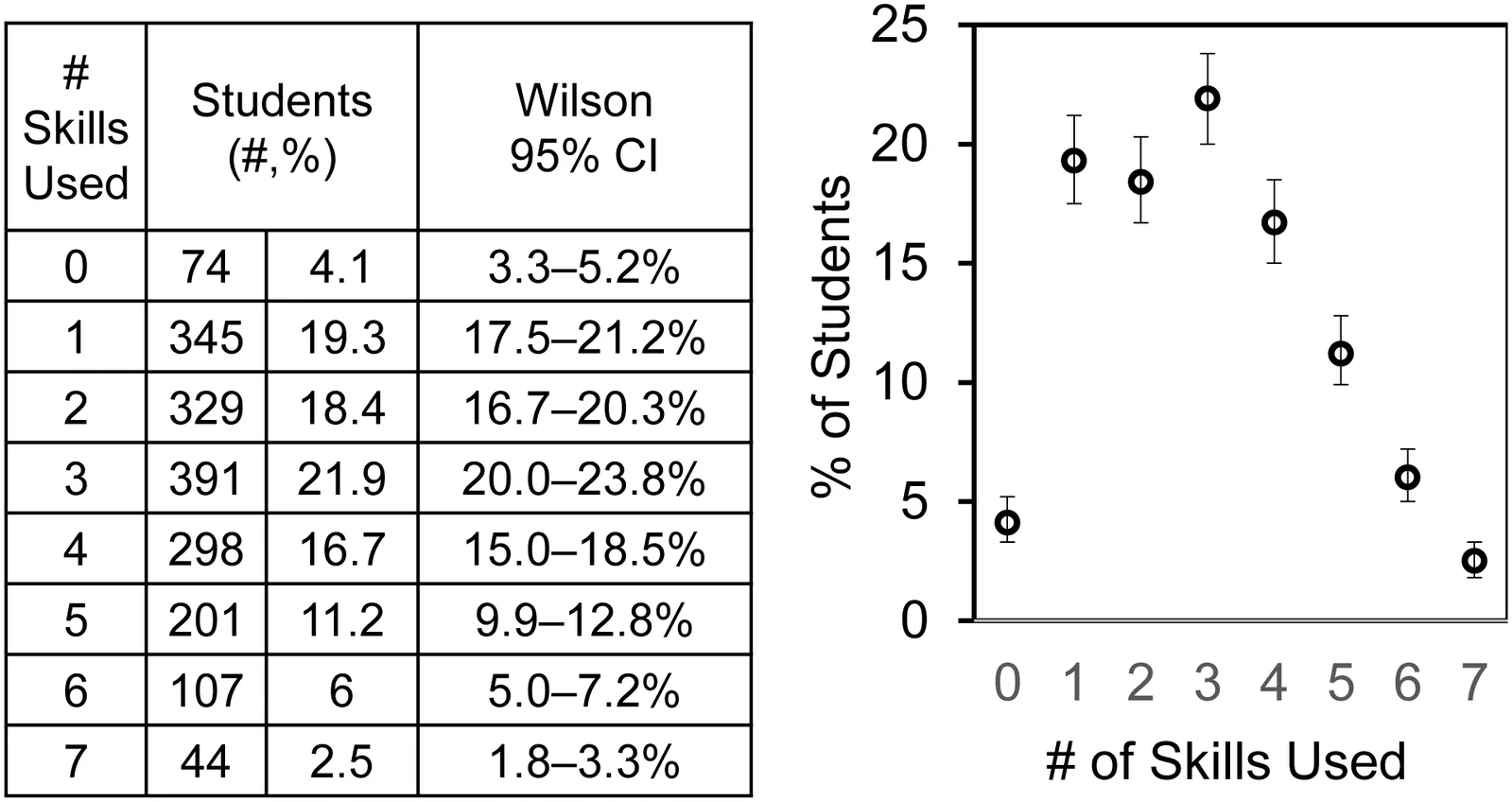
Overall frequency of skill use. Error bars indicate upper and lower limits of Wilson 95% confidence intervals. Mean number of skills used = 2.99.

Fig 2 shows the overall extent of communication and presentation skill use. The number of skills used ranged from zero (4.1% of the students) to all seven (2.5% of the students). The mean number of skills used per student was 2.99.

Subsequent figures show how individual skills differed according to student demographics, help received, and SEF outcomes. These analyses identify associations between communication practices and outcomes but cannot determine causality.

### Demographics

**Fig 3:**
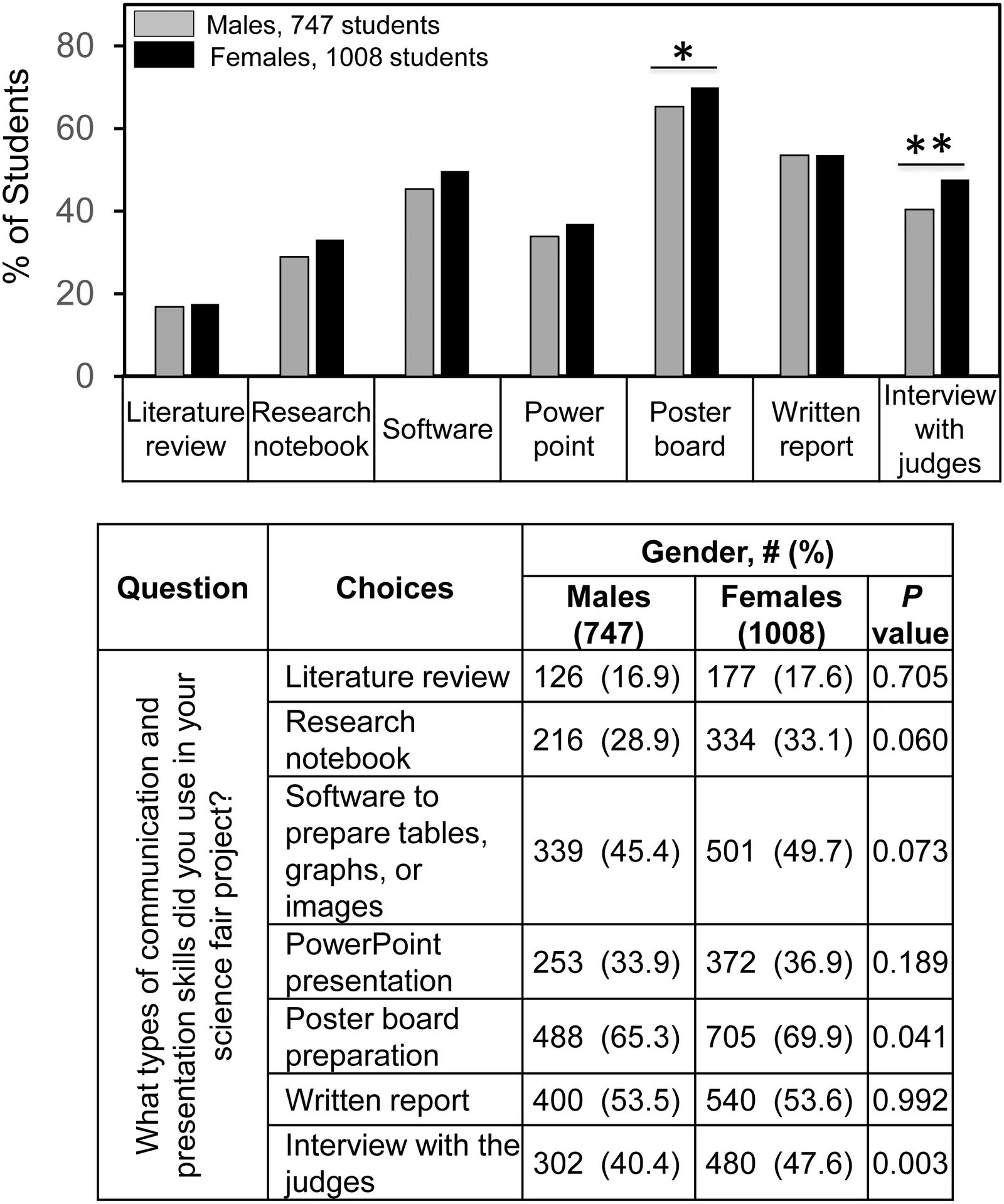
Skill use and gender. Differences depending on gender were evaluated using Pearson's chi-square test. *P < 0.05; **P < 0.01; ***P < 0.001. 34 students did not report gender.

**Fig 4:**
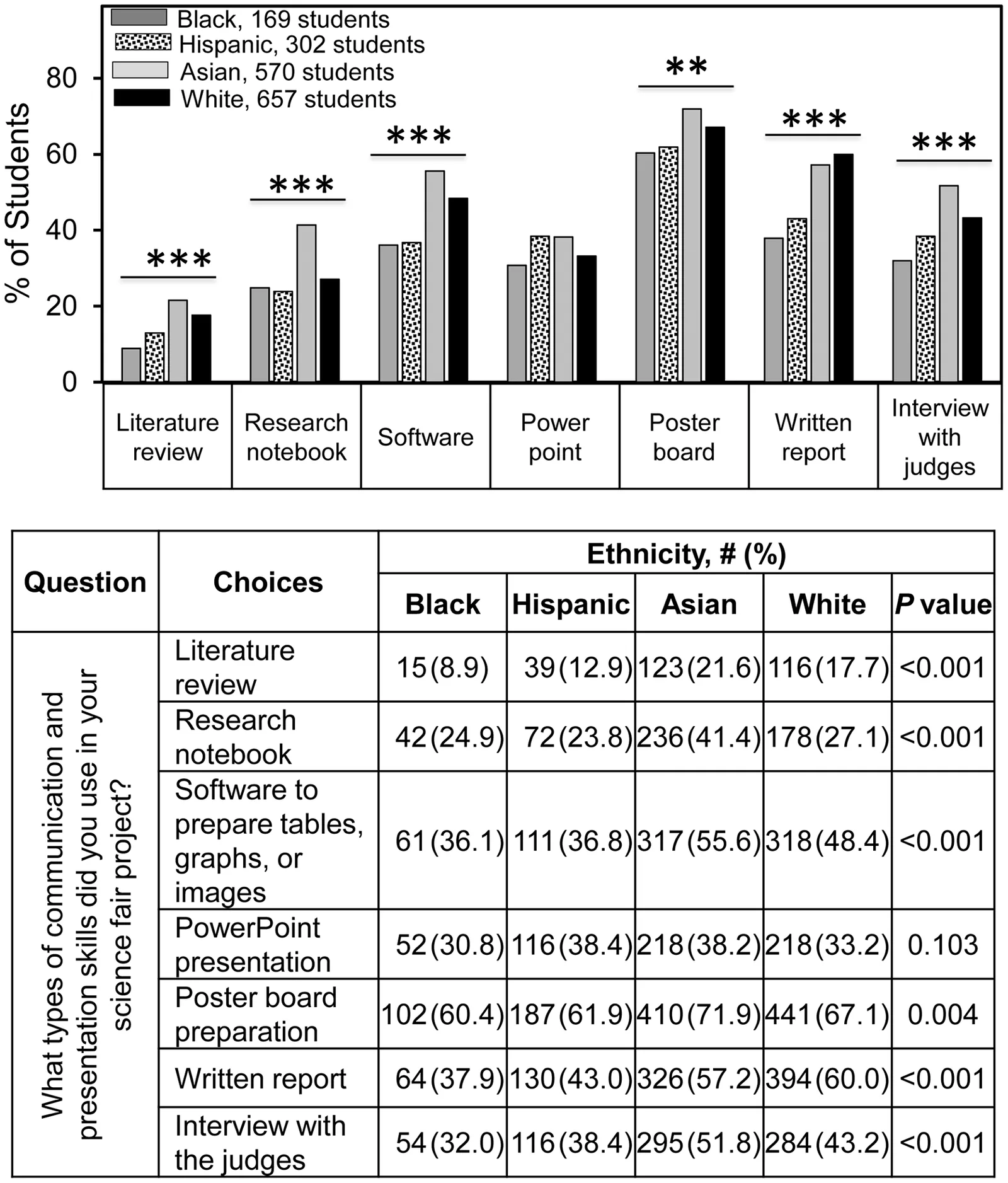
Skill use and ethnicity. Differences depending on ethnic group were evaluated using Pearson's chi-square test. *P < 0.05; **P < 0.01; ***P < 0.001. 91 students did not report ethnicity.

In Figs 3 and 4, survey results were analyzed to determine if differences in particular skill use were associated with gender or ethnicity. Fig 3 shows survey responses based on gender. Overall, use of communication and presentation skills showed only small gender differences in the various categories. Females reported slightly higher percentages in most categories.

Fig 4 shows survey responses based on ethnicity. Unlike gender, greater variation was observed across ethnic groups. Asian students reported higher percentages of skill use; White students were intermediate; and Hispanic and Black students were lower. The most notable differences were higher use by Asian students for literature review, research notebook and interview with the judges; and higher use by Asian and White students for software and written report. Use of PowerPoint presentation and poster board preparation were similar for all groups.

### Help students received

**Fig 5:**
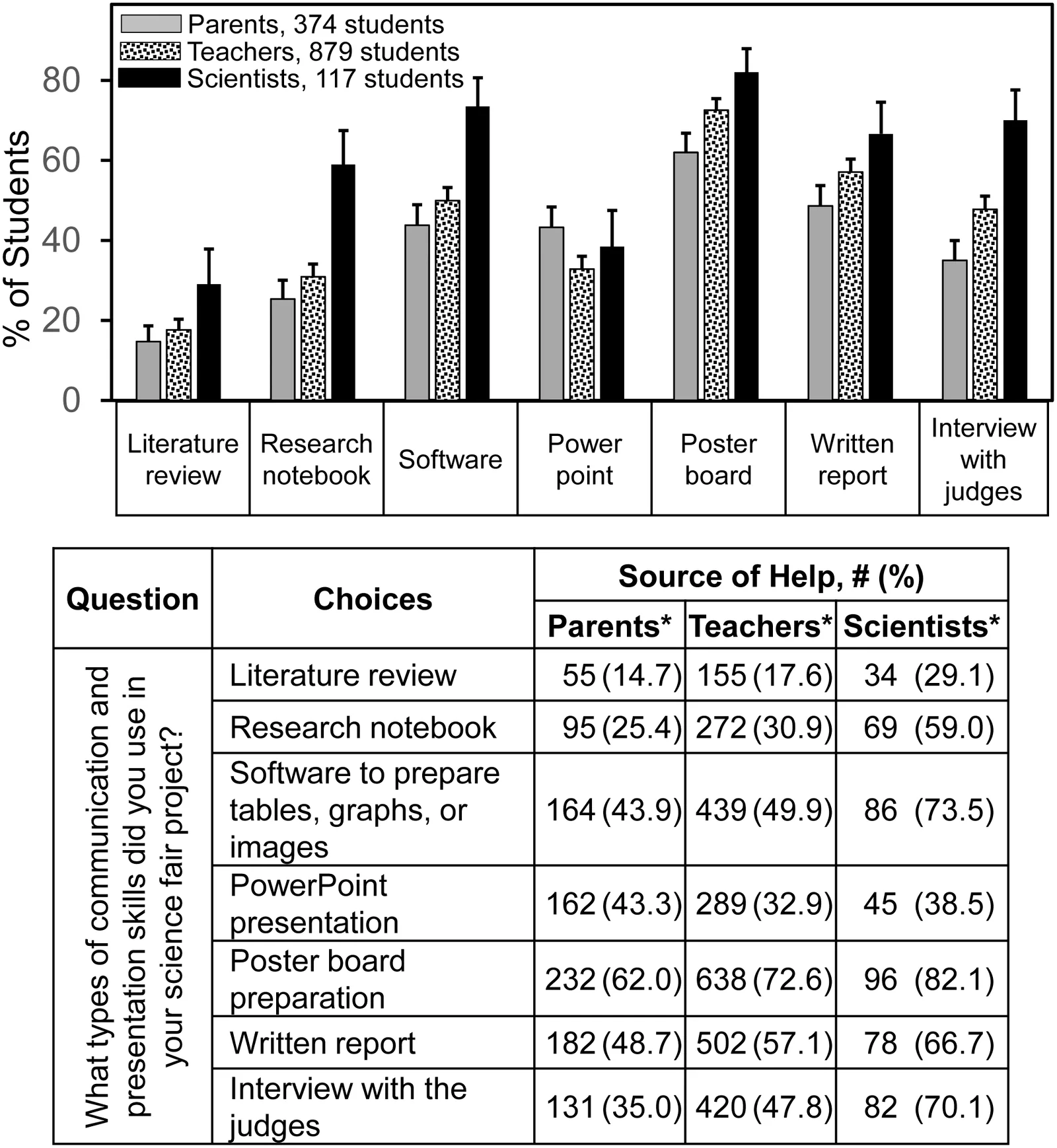
Skill use and help from parents (only), teachers (could include parents), and scientists (could include parents and teachers). Solid bars show the percentage of students; error bars show the upper limits of the 95% Wilson confidence intervals. Complete Wilson 95% confidence intervals can be found in S1 Table. 419 students did not answer the question about sources of help.

**Fig 6:**
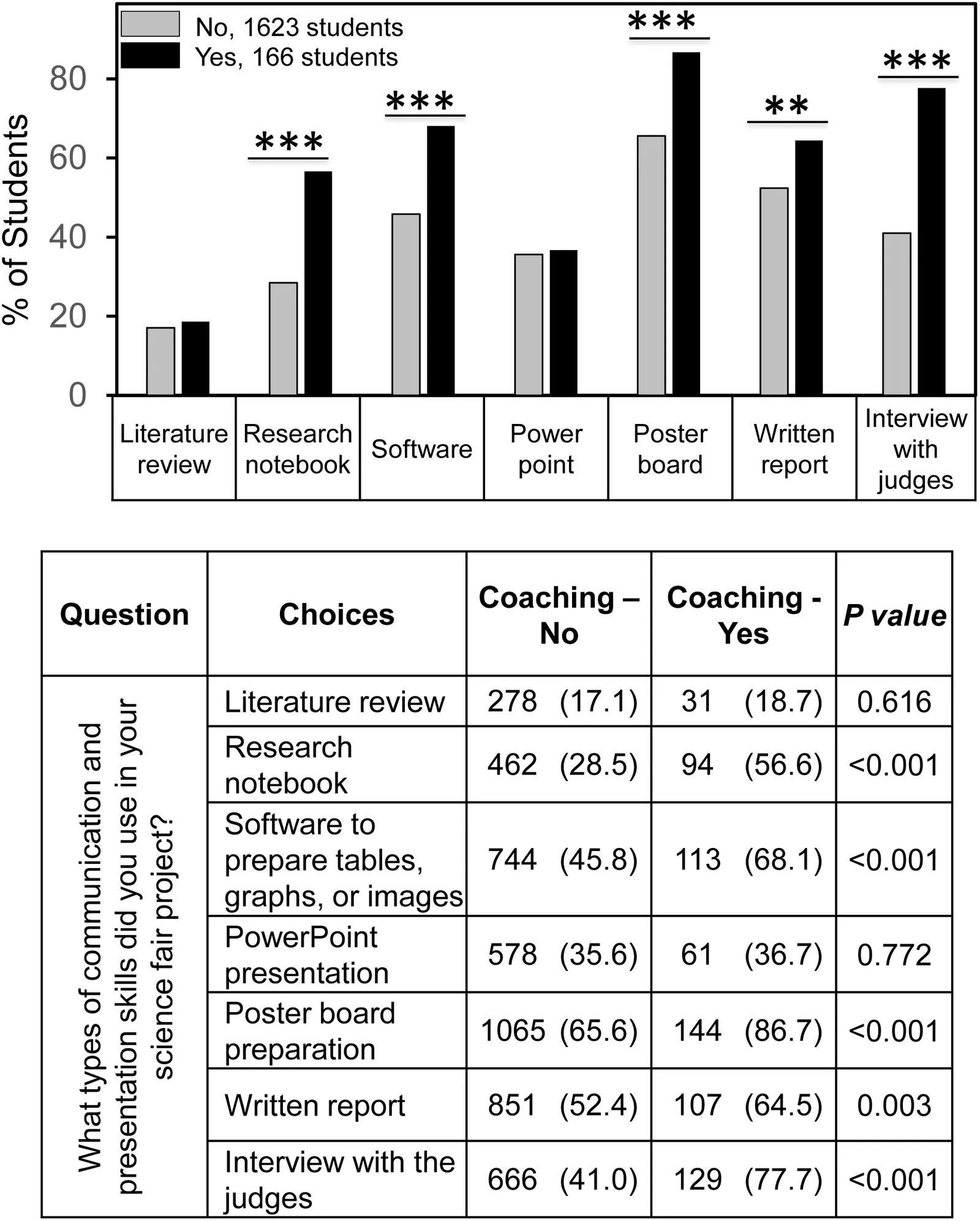
Skill use and coaching for the interview. Differences depending on coaching were evaluated using Pearson's chi-square test. *P < 0.05; **P < 0.01; ***P < 0.001.

In Figs 5 and 6, survey results were analyzed to determine if differences in particular skill use were associated with types of help that students received. Fig 5 shows survey responses based on help from parents (alone), teachers (could also include parents), and scientists (could also include parents and teachers). Except for use of PowerPoint presentation, students receiving help from scientists were more likely to report a higher percentage of skill use followed by students receiving help from teachers and then from parents. In the case of use of a research notebook, students with help from scientists were twice as likely (60% vs. 30%) to report use of the skill.

Fig 6 shows survey responses based on whether students received coaching for the interview. Except for literature review and use of PowerPoint presentation, coaching for the interview was associated with an increase in skill use. As in the case of help from scientists, the largest difference was in reported use of a research notebook (57% vs. 29%). Also, students who received coaching for the interview reported a higher likelihood of interviews with judges (78% vs. 41%).

### SEF outcomes and skill use

**Fig 7:**
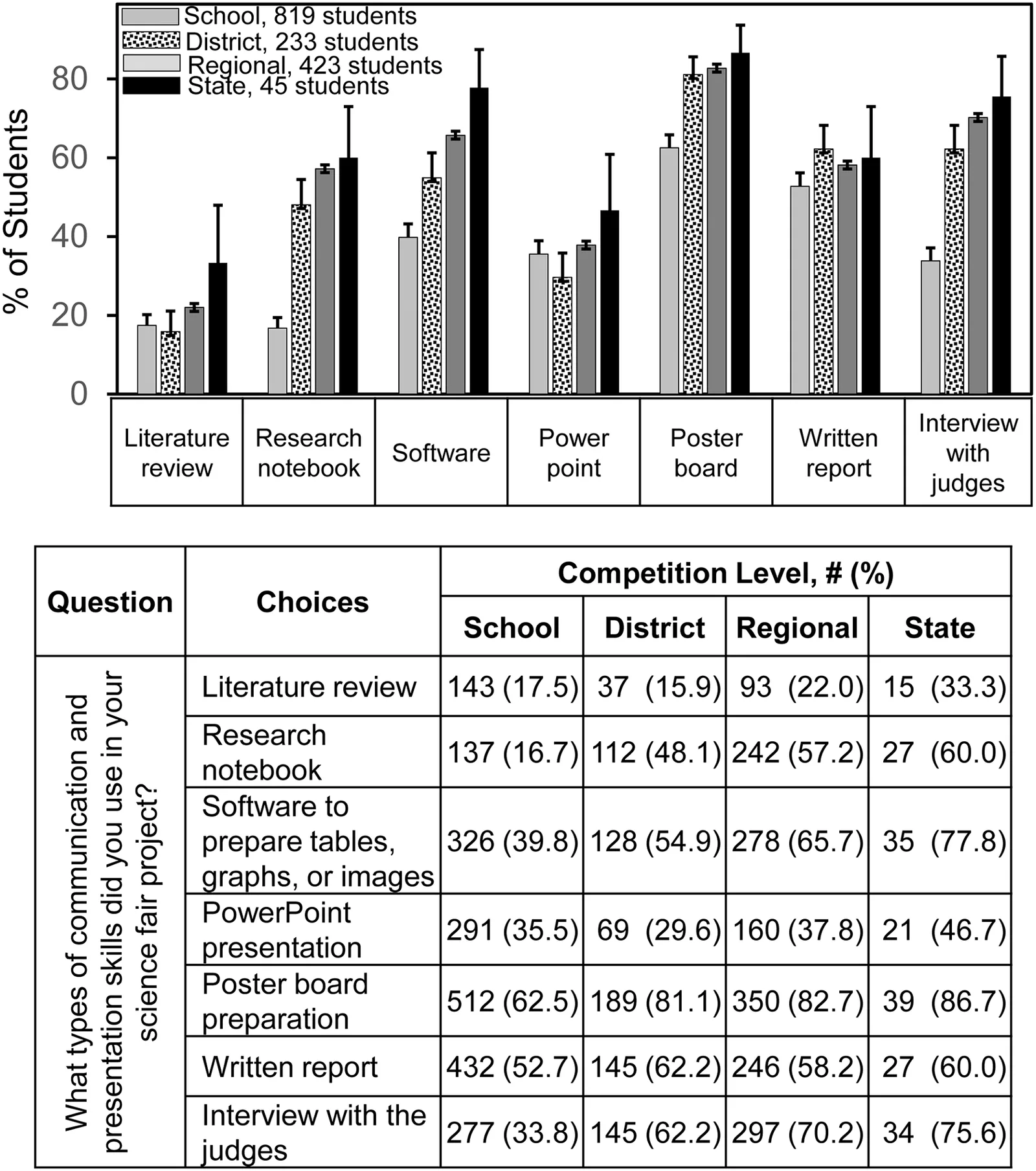
Skill use and level of SEF competition: school (only); district (could include school); region (could include school and/or district); state (could include school, district and/or region). Solid bars show the percentage of students; error bars show the upper limits of the 95% Wilson confidence intervals. Complete Wilson 95% confidence intervals can be found in S1 Table. 269 students did not report level of SEF competition.

Fig 7 shows skill use at progressively higher levels of SEF competition. In this comparison, students could participate in SEF competitions ranging from school to state level. We don’t know the SEF competition levels to which each individual student had access. The results shown are for the number of skills used by students at the highest level of SEF competition in which they reported competing. The average number of skills used by students increased with higher levels of competition - school (2.6), district (3.5), regional (3.9) and state (4.4). One noticeable trend was that students in the school only group reported especially lower percentages of use of research notebooks and interview with the judges compared to students in the other competition levels, whereas use of written reports was similar across competition levels.

**Fig 8:**
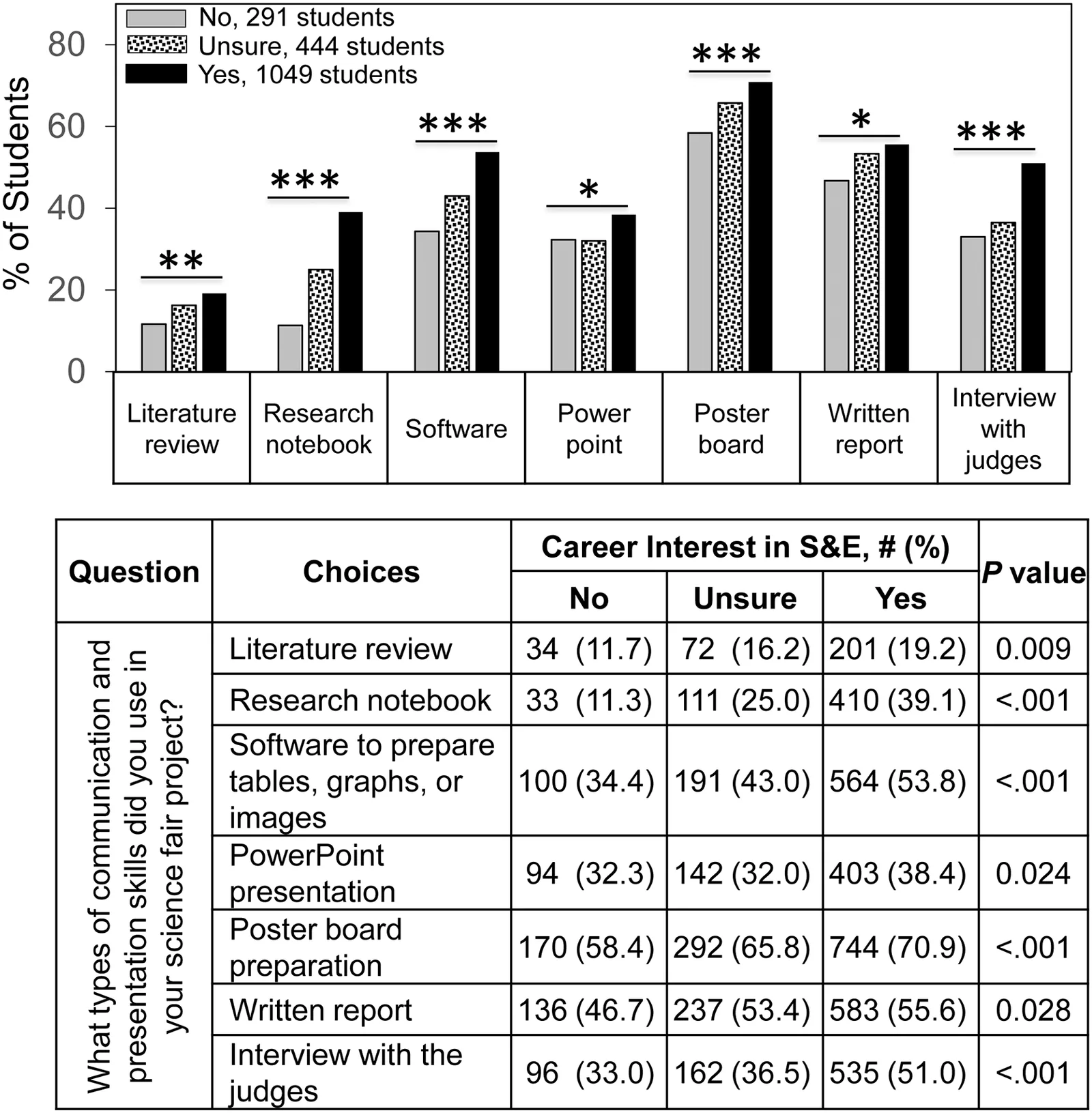
Skill use and student interest in an S&E career. Differences between groups were evaluated using Pearson's chi-square test. *P < 0.05; **P < 0.01; ***P < 0.001. 5 students did not answer the question about interest in an S&E career.

Fig 8 shows use of communication and presentation skills in relation to whether students were interested in a career in S&E. The overall trend was for students who selected having a career interest to report greater skill use compared to students with unsure or no interest in an S&E career. Four of the skills about which we asked students showed a strong association (*P* < .001) with greater interest – research notebook; software to prepare tables, graphs and images; poster board preparation; and interview with the judges. Of this group, use of a research notebook stood out as more than three times as likely to be used by students interested in an S&E career.

**Fig 9:**
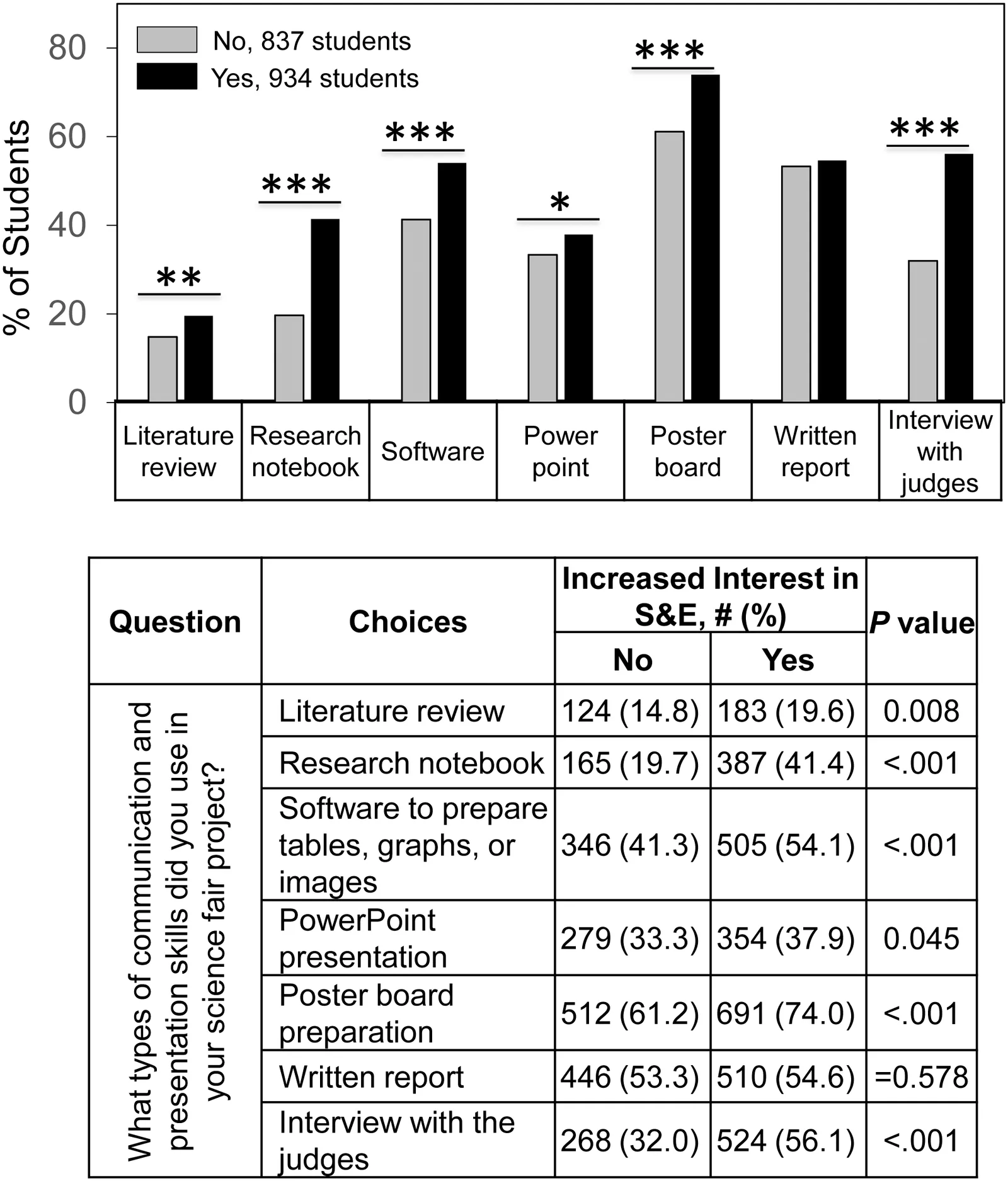
Skill use and whether SEF participation increased student interest in S&E. Differences between groups were evaluated using Pearson's chi-square test. *P < 0.05; **P < 0.01; ***P < 0.001. 18 students did not answer the question about whether SEF participation increased their interest in S&E.

Fig 9 shows use of communication and presentation skills in relation to whether students indicated that participation in SEFs increased their interest in S&E. Similarly to the results regarding interest in an S&E career, four of the skills about which we asked students showed a strong association (*P* < .001) with increased interest – research notebook; software to prepare tables, graphs and images; poster board preparation; and interview with the judges. Use of a research notebook and interview with judges stood out and were twice as likely to be used by students who indicated that SEF participation increased their interest in S&E.

## Discussion

Communication and presentation skills are integral parts of scientific inquiry and involved in choosing research questions, documenting evidence, interpreting findings, and reporting conclusions. In this paper, we present findings based on quantitative answers by 1789 students who participated in our SEF surveys during 2021−22 and 2022−23 and answered the question “What types of communication and presentation skills did you use in your science fair project?” The possible answers were *literature review; research notebook; software to prepare tables, graphs, or images; written report; poster board preparation; PowerPoint presentation; and interview with the judges*.

The possible answers connect with different phases of scientific inquiry. Literature review and research notebook are part of developing research questions and data collection. Software to prepare tables, graphs, or images, and PowerPoint presentation contribute to analyzing research and developing a presentation. Written reports, poster board preparation, and interviews with the judges provide the opportunity to present the findings. Among these, only poster board preparation and interview with the judges are skills explicitly included as part of typical SEF scoring criteria. Judges are advised to examine the student notebook but not to score it [46].

On average, students indicated they used three skills, which most often were those included in SEF project scoring -- poster board preparation and interview with the judges. These two options were selected more frequently than literature review and research notebook although the latter two options are implicit in scientific inquiry. As SEFs are now scored, the latter skills can only be assessed indirectly through inclusion in sections of the poster board or written report or as part of the interview. Currently, presentation of the results appears to get the most attention.

One possibility is that students who already are interested in STEM may be more likely to engage with a scientist, use research notebooks, or complete a literature review. Explicitly including literature review and research notebooks in SEF scoring might encourage students to give these practices greater attention. However, adding these additional requirements also might create a barrier to participation, particularly for students who are less certain of their interest in science or who have fewer educational resources. Our findings cannot distinguish between these possibilities.

Demographic studies showed few gender differences between students. However, we observed ethnicity-dependent differences. Asian students reported a higher percentage of skill use in most categories. Overall skill use per student was highest for Asian students (3.4) followed by White students (3.0) and then Hispanic and Black students (2.6 and 2.3). Since the differences between ethnic groups were evident for interview with the judges but not poster board preparation, one interpretation of this finding is that students in all groups had the opportunity to present their work by poster even if they didn’t get an interview with judges.

Previously we found that Asian and Hispanic students indicated more often than Black and White students that SEF participation increased their interest in S&E [41, 42] and made more free text positive comments about their SEF experiences [37]. The present study suggests that differences in the students’ uses of communication and presentation skills cannot account for the differences in the students’ attitudes towards SEF experience that we reported previously.

More than 50% of students receive help from parents and teachers. Less than 10% receive help from scientists. However, compared to other students, those who received help from scientists were much more likely to report use of a literature review, research notebook, and software, all typical features of scientific inquiry. Help from teachers also had an impact but mostly on the skills involved in presentation of the research rather than inquiry itself. Students who received coaching for the interview also indicated greater use of a research notebook and software. These differences provide additional support for the conclusion that help from scientists and coaching should be expanded as much as possible to advise students about their SEF projects [32–34,41,52].

The finding that the average number of skills used by students increased with higher levels of competition - school (2.6), district (3.5), regional (3.9) and state (4.4) – is consistent with greater student engagement with scientific inquiry. The trend was not just overall but for almost every type of skill except written report. However, these differences might reflect not only the students’ abilities but also in part their school’s involvement. That is, not all schools or school districts have the financial and other resources to make it possible for students to participate in SEFs beyond the school only level. The findings suggest that for school only SEFs, use of written reports may provide an opportunity for students to present their SEF research even if an interview to verbally present the work with judges is unavailable.

Whether or not students indicated that they are interested in a career in S&E and whether SEF participation increased their interest in S&E are key indicators of successful SEF outcomes. Four of the skills about which we asked students showed a strong association (*P* < .001) with successful outcomes – research notebook; software to prepare tables, graphs and images; poster board preparation; and interview with the judges. Of this group, use of a research notebook stood out as more than three times as likely to be used by students interested in an S&E career and twice as likely to be used by students who indicated that SEF participation increased their interest in S&E. Ironically, even though students do not select research notebooks as one of the more frequently used skills in SEFs and are not scored on this skill explicitly, the science education literature has long emphasized the value of research notebooks to help students engage in reflection, interpretation, and conceptual understanding of their work [53–56] as well as improving literacy overall [57–59].

Several limitations of our study are worth noting. One is that we treat the Scienteer SEF population as a national group. However, it should be recognized that these students may not be truly representative of a national sample since they come from only 7 U.S. states and only attend high schools where SEFs are available. In addition, we cannot be sure that the 3% response rate of survey respondents is representative of the overall high school student population participating in SEFs. Also, using PowerPoint as a response option may have missed students who made computer-generated slides using Canva or other software. Finally, we cannot be sure that students mean the same thing when they choose the different skills. For instance, in Fig 2 it seems unlikely that a science fair project could be completed without any of the skills listed.

In conclusion, our findings help understand the ways in which communication and presentation practices were associated with SEF participation and outcomes and how these practices can be encouraged in different ways by engagement with teachers, scientists, and coaching. Surprisingly, inquiry practices of literature review and research notebook, which provide the underlying framework for developing and carrying out scientific inquiry, are least selected by students, perhaps because of how SEFs are scored. More than any other skill, use of research notebooks was associated with positive SEF outcomes.

## Supporting information

S1 File Human Research Approvals. (PDF)

S2 File Survey. (PDF)

S3 File Data Set. (XLSX)

S1 Table Confidence Intervals. (DOCX)

## References

[1] National Research Council. A Framework for K-12 Science Education: Practices, Crosscutting Concepts, and Core Ideas. Washington, D.C.: National Academies Press. 2012.

[2] NGSS Lead States. Next Generation Science Standards for States, By States. Volume 1: The Standards—Arranged by Disciplinary Core Ideas and by Topics. Washington, D.C.: National Academies Press. 2013.

[3] Furtak EM, Penuel WR. Coming to terms: addressing the persistence of “hands‐on” and other reform terminology in the era of science as practice. Science Education. 2018;103(1):167–86. doi: 10.1002/sce.21488

[4] Lederman NG. Contextualizing the relationship between nature of scientific knowledge and scientific inquiry. Sci & Educ. 2019;28(3–5):249–67. doi: 10.1007/s11191-019-00030-8

[5] Osborne J. Teaching scientific practices: meeting the challenge of change. Journal of Science Teacher Education. 2014;25(2):177–96. doi: 10.1007/s10972-014-9384-1

[6] Plans science club for school pupils. The New York Times. 1932.

[7] Bencze JL, Bowen GM. A national science fair: exhibiting support for the knowledge economy. International Journal of Science Education. 2009;31(18):2459–83. doi: 10.1080/09500690802398127

[8] Janštová V, Kotrčová E, Matějková T. Key aspects of successful science fair projects. International Journal of Science Education. 2024;:1–17. doi: 10.1080/09500693.2024.2364956

[9] Lachebo GC, Thuo MW, Labiso TO, Demissie EB. Students’ readiness to participate in science project competitions: Views from secondary schools in southern Ethiopia. Social Sciences & Humanities Open. 2024;10:101033. doi: 10.1016/j.ssaho.2024.101033

[10] So WWM. Does computation technology matter in science, technology, engineering and mathematics (STEM) projects?. Research in Science & Technological Education. 2021;41(1):232–50. doi: 10.1080/02635143.2021.1895099

[11] Paul J, Lederman NG, Groß J. Learning experimentation through science fairs. International Journal of Science Education. 2016;38(15):2367–87. doi: 10.1080/09500693.2016.1243272

[12] Konur KB, Yazici A. Evaluation of 4006 TUBITAK science fairs in terms of science teachers. eqr. 2022;5(3). doi: 10.31014/aior.1993.05.03.537

[13] Rahman N. Effectiveness of a project based learning model integrated with sasambo local wisdom to enhance students’ scientific attitudes. JIPI. 2025. doi: 10.24815/jipi.v9i1.42626

[14] Ndlovu M. Learner perceptions of inquiry in science fair projects. International Journal of Educational Sciences. 2015.

[15] Muniz R. Development of a responsive web tool for managing scientific project submissions. International Journal of Advanced Academic Studies. 2025. doi: 10.33545/27068919.2025.v7.i4b.1430

[16] Marchak D. Exploring motivational mechanisms to learn in a national chemistry projects competition. Disciplinary and Interdisciplinary Science Education Research. 2026. doi: 10.1186/s43031-025-00152-w

[17] Society for Science. About ISEF 2026. https://www.societyforscience.org/isefpartnership/. Accessed 2026 May 16.

[18] Kook JF, DeLisi J, Fields ET, Levy AJ. Approaches for conducting middle school science fairs: a landscape study. Science Educator. 2020;27(2):71–80.

[19] Gao S. What science fairs reveal about STEM learning. Education Sciences. 2026. doi: 10.3390/educsci16030482

[20] Burgin SR, Sadler TD. Learning nature of science concepts through a research apprenticeship program: a comparative study of three approaches. J Res Sci Teach. 2015;53(1):31–59. doi: 10.1002/tea.21296

[21] Houseal AK, Abd-El-Khalick F, Destefano L. Impact of a student-teacher-scientist partnership on students’ and teachers’ content knowledge, attitudes toward science, and pedagogical practices. J Res Sci Teach. 2013;51(1):84–115. doi: 10.1002/tea.21126

[22] Andersson J, Schaben C, Buhs E, Grandgenett N. Phenomenology of secondary students’ experiences in out-of-school time science research. Science Educator. 2021;28(1):30–9.

[23] Godse S, Sapar T, Amacher JF. An idea to explore: Engaging high school students in structure-function studies of bacterial sortase enzymes and inhibitors - a comprehensive computational experimental pipeline. Biochem Mol Biol Educ. 2023;51(6):606–15. doi: 10.1002/bmb.21769 37462254

[24] Mims PJ, Lee LE, Kuldell N, Franklin C. Strengthening the STEM pipeline: impact of project-based synthetic biology program on high school students’ science identity and competency. Front Educ. 2025;9. doi: 10.3389/feduc.2024.1493356

[25] Emery K, Harlow D, Whitmer A, Gaines S. Confronting ambiguity in science. The Science Teacher. 2015;:36–9.

[26] Rodenbusch SE, Hernandez PR, Simmons SL, Dolan EL. Early engagement in course-based research increases graduation rates and completion of science, engineering, and mathematics degrees. CBE Life Sci Educ. 2016;15(2):ar20. doi: 10.1187/cbe.16-03-0117 27252296 PMC4909342

[27] Hanauer DI, Graham MJ, Betancur L, Bobrownicki A, Cresawn SG, et al. An inclusive Research Education Community (iREC): impact of the SEA-PHAGES program on research outcomes and student learning. Proc Natl Acad Sci U S A. 2017;114(51):13531–6. doi: 10.1073/pnas.1718188115 29208718 PMC5754813

[28] Beier ME, Kim MH, Saterbak A, Leautaud V, Bishnoi S, Gilberto JM. The effect of authentic project‐based learning on attitudes and career aspirations in STEM. J Res Sci Teach. 2018;56(1):3–23. doi: 10.1002/tea.21465

[29] Kutluk H, Jaworski D, Zheng H, Krajka V, Constantinou I. Designed-to-fail: using structured failure in laboratory courses as a tool for nature of science education. Education Sciences. 2025;15(9):1115. doi: 10.3390/educsci15091115

[30] Mernoff B, Aldous AR, Wasio NA, Kritzer JA, Sykes ECH, O’Hagan K. A Reverse Science Fair that Connects High School Students with University Researchers. J Chem Educ. 2017;94(2):171–6. doi: 10.1021/acs.jchemed.6b00111

[31] Lakin JM, Ewald ML, Hardy EE, Cobine PA, Marino JG, Landers AL, et al. Getting everyone to the fair: supporting teachers in broadening participation in science and engineering fairs. J Sci Educ Technol. 2021;30(5):658–77. doi: 10.1007/s10956-021-09910-7 33758488 PMC7970784

[32] Koomen MH, Hedenstrom MN, Moran MK. Rubbing elbows with them: building capacity in STEM through science and engineering fairs. Science Education. 2021;105(3):541–79. doi: 10.1002/sce.21615

[33] Stray S, Gordy X, Sullivan D, Bender S, Cook C, McKone K, et al. Base pair: 28 years of sustained high school biomedical research mentorship driving health sciences career progression. Journal of STEM Outreach. 2020;3(3):1–10.

[34] Todd C. Collaborations between under-resourced high school students and STEM professionals to increase participation in science and engineering fairs. EJEDU. 2022;3(1):1–6. doi: 10.24018/ejedu.2022.3.1.205

[35] Hougham R, Bessler B, Zocher J, Sams W. Engaging diverse communities in a community science fair. THST. 2023;46(2):6–11. doi: 10.14434/thst.v46i2.36529

[36] Chiu TKF. Using self-determination theory (SDT) to explain student STEM interest and identity development. Instr Sci. 2023;52(1):89–107. doi: 10.1007/s11251-023-09642-8

[37] Grinnell F, Dalley S, Reisch J. High school science fair: what students say-mastery, performance, and self-determination theory. PLoS One. 2025;20(6):e0325283. doi: 10.1371/journal.pone.0325283 40560848 PMC12193632

[38] Andersson J. Using constructivist grounded theory to understand why female secondary students engage in scientific research. AM J QUALITATIVE RES. 2025;9(4):191–218. doi: 10.29333/ajqr/17134

[39] Cullen S, Oppenheimer D. Choosing to learn: the importance of student autonomy in higher education. Sci Adv. 2024;10(29):eado6759. doi: 10.1126/sciadv.ado6759 39018403 PMC466944

[40] Ryan RM, Deci EL. Intrinsic and extrinsic motivation from a self-determination theory perspective: Definitions, theory, practices, and future directions. Contemporary Educational Psychology. 2020;61:101860. doi: 10.1016/j.cedpsych.2020.101860

[41] Grinnell F, Dalley S, Reisch J. High school science fair: ethnicity trends in student participation and experience. PLoS One. 2022;17(3):e0264861. doi: 10.1371/journal.pone.0264861 35320299 PMC8942272

[42] Grinnell F, Dalley S, Reisch J. High school science fair: school location trends in student participation and experience. PLoS One. 2023;18(9):e0291049. doi: 10.1371/journal.pone.0291049 37695794 PMC10495023

[43] Grinnell F, Dalley S, Reisch J. High school science fair: experiences of two groups of undergraduate bioscience students. PLoS One. 2021;16(6):e0252627. doi: 10.1371/journal.pone.0252627 34086767 PMC8177480

[44] Grinnell F, Dalley S, Shepherd K, Reisch J. High school science fair: student opinions regarding whether participation should be required or optional and why. PLoS One. 2018;13(8):e0202320. doi: 10.1371/journal.pone.0202320 30096184 PMC6086443

[45] Grinnell F, Dalley S, Reisch J. High school science fair: positive and negative outcomes. PLoS One. 2020;15(2):e0229237. doi: 10.1371/journal.pone.0229237 32053697 PMC7018130

[46] Society for Science. Grand Award Judging Criteria 2026. https://www.societyforscience.org/isef/grand-award/criteria/

[47] Harris PA, Taylor R, Thielke R, Payne J, Gonzalez N, Conde JG. Research electronic data capture (REDCap)--a metadata-driven methodology and workflow process for providing translational research informatics support. J Biomed Inform. 2009;42(2):377–81. doi: 10.1016/j.jbi.2008.08.010 18929686 PMC2700030

[48] Kent R, Brandal H. Improving Email response in a permission marketing context. International Journal of Market Research. 2003;45(4):1–13. doi: 10.1177/147078530304500404

[49] Van Mol C. Improving web survey efficiency: the impact of an extra reminder and reminder content on web survey response. International Journal of Social Research Methodology. 2016;20(4):317–27. doi: 10.1080/13645579.2016.1185255

[50] Shih T-H, Xitao Fan. Comparing response rates from web and mail surveys: a meta-analysis. Field Methods. 2008;20(3):249–71. doi: 10.1177/1525822x08317085

[51] Grinnell F, Dalley S, Shepherd K, Reisch J. High school science fair and research integrity. PLoS One. 2017;12(3):e0174252. doi: 10.1371/journal.pone.0174252 28328976 PMC5362261

[52] Minocha T, Bhagatwala T, Mirzoyan G, McDowell G, Fankhauser SC. Empowering future scientists: mentors employ various strategies to engage students in professional science disciplinary literacy practices. Discip Interdscip Sci Educ Res. 2025;7(1). doi: 10.1186/s43031-025-00124-0

[53] Ruiz-Primo MA, Li M. On the validity of science notebook assessments. Educational Assessment. 2004.

[54] Keys CW. Using the science writing heuristic to promote learning from laboratory activities. Journal of Research in Science Teaching. 1999.

[55] Hand B. The science writing heuristic. Int J Sci Educ. 2004.

[56] Akkus R. Effectiveness of the science writing heuristic. International Journal of Science Education. 2007.

[57] Huerta M, Garza T. Academic language and conceptual development through science notebooks for english language learners. Journal of Science Education. 2016.

[58] Cervetti G. Integrating literacy and science instruction. Reading Research Quarterly. 2012.

[59] Lee O. Science and literacy integration for English language learners. Journal of Research in Science Teaching. 2005.

