# Supplementary material for: High school science fair: Characterizing student communication and presentation practices and their association with SEF outcomes
Source: PLoS One. 2026 Sep 11;21(9):e0357589. doi: 10.1371/journal.pone.0357589 (PMC13567831; doi:10.1371/journal.pone.0357589)
Supplement: Supplementary file 1 [file pone.0357589.s001.pdf]

UT SOUTHWESTERN MEDICAL CENTER — eIRB System

From:	William Engle<br>Institutional Review Board Chairperson<br>IRB - 8843
To:	Frederick Grinnell
Date:	August 6, 2014
Re:	Study Approval
IRB Number:	STU 072014-076
Title:	Research Practices in Secondary School Science Fair
Documents:	Protocol and All Smart Form Attachments

The UT Southwestern Institutional Review Board (IRB) reviewed the above-referenced research study via an expedited review procedure on August 6, 2014 in accordance with 45 CFR 46.110(a)-(b)(1). Having met all applicable requirements, the research study is approved for continuation for a period of 12 months. The approval period for this research study begins on August 6, 2014 and lasts until August 5, 2015.

Documentation of consent was waived in accordance with 45 CFR 46.117(c).

The research study cannot continue beyond the approval period without continuing review and approval by the IRB. In order to avoid a lapse in IRB approval, the Principal Investigator must apply for continuing review of th protocol and related documents before the expiration date. A reminder will be sent to you approximately 90 days prior to expiration of research study approval.

The approved number of subjects to be enrolled is 2500. The IRB considers a subject to be enrolled once s/h verbally agrees to participate in the study. If additional subjects are needed, you first must obtain permissio from the IRB to increase the sample size.

If you have any questions related to this approval letter or about IRB policies and procedures, please telephon the IRB Office at 214-648-3060.

Human Research Protection Program

APPROVAL

February 25, 2026

Frederick Grinnell
UT Southwestern
Cell Biology
FREDERICK.GRINNELL@UTSouthwestern.edu

Dear Dr. Frederick Grinnell

On February 25, 2026, the IRB reviewed the following submission:

Type	Description
Type of Review:	Continuing Review
Title:	REINVENTING SCIENCE FAIRS
Investigator:	Frederick Grinnell
IRB ID:	STU 072014-076
Submission ID:	CR12-STU 072014-076
Funding:	• Internal - Departmental
Reviewing IRB:	UT Southwestern IRB
REVIEW DECISION:	Approved [IRB Determination]
Documents Reviewed:	None

The IRB approved the study on 2/25/2026.

IRB Expiration Date: 4/18/2027

A Continuing Review (CR) submission is required no later than **30 days before the study expiration date**. Failure to submit by this deadline may result in study expiration and suspension of research activities.

All proposed modifications to the study must receive IRB approval **before implementation**. The only exception is when a change is made to eliminate an **immediate hazard to participants**. Such emergency actions are

considered **emergency violations** and must be submitted promptly to the IRB as a **Reportable Event**.

Investigators are required to continue monitoring the study and to promptly report **Unanticipated Problems Involving Risks to Subjects or Others (UPIRSO)** as well as any instances of **noncompliance** in accordance with IRB policy. If changes are made to this research which may affect this determination, submit those changes to HRPP for review.

If changes are made to this research which may affect this determination, submit those changes to HRPP for review.

General Instructions

To maintain IRB approval in good standing, please observe the following requirements:

1. Obtain prior IRB approval for any modifications including addition of new recruiting materials, changes i research personnel or site location, sponsor amendments or other changes to the protocol or associate documents. Only those changes that are necessary to avoid an immediate apparent hazard to a subje may be implemented without prior IRB approval.
2. Report all adverse events, protocol violations, and study closures promptly to the IRB.
3. Make study records available for inspection. All research-related records and documentation may b inspected by the IRB for the purpose of ensuring compliance with UT Southwestern policies an procedures and federal regulations governing the protection of human subjects. The IRB has authorit to suspend or terminate its approval if applicable requirements are not strictly adhered to by all researc study personnel.
4. When enrolling subjects who do not speak or read English, a bilingual translator must be available to facilitate communications between research personnel and a subject.

Warning: This is a private message for authorized UT Southwestern employees only. If the reader of this message is not the intended recipient are hereby notified that any dissemination, distribution or copying of this information is STRICTLY PROHIBITED.

University of Texas Southwestern Medical Center
Institutional Review Board

5323 Harry Hines Boulevard
Dallas, Texas 75390-8843
Room C1.206
phone: 214-648-3060
fax: 214-648-2171
