# Supplementary material for: High school science fair: Characterizing student communication and presentation practices and their association with SEF outcomes
Source: PLoS One. 2026 Sep 11;21(9):e0357589. doi: 10.1371/journal.pone.0357589 (PMC13567831; doi:10.1371/journal.pone.0357589)
Supplement: Supplementary file 3 [file pone.0357589.s004.docx]

Wilson 95% confidence intervals for Figure 5.

| **Communication skill** | **Parents % (95% CI)** | **Teachers % (95% CI)** | **Scientists % (95% CI)** |
| --- | --- | --- | --- |
| Literature review | 14.7 (11.5–18.7) | 17.6 (15.3–20.3) | 29.1 (21.6–37.8) |
| Research notebook | 25.4 (21.3–30.0) | 30.9 (28.0–34.1) | 59.0 (49.9–67.5) |
| Software | 43.9 (38.9–48.9) | 49.9 (46.6–53.2) | 73.5 (64.9–80.7) |
| PowerPoint | 43.3 (38.4–48.4) | 32.9 (29.9–36.1) | 38.5 (30.1–47.5) |
| Poster board | 62.0 (57.0–66.8) | 72.6 (69.5–75.4) | 82.1 (74.1–88.0) |
| Written report | 48.7 (43.6–53.7) | 57.1 (53.8–60.3) | 66.7 (57.7–74.6) |
| Interview with judges | 35.0 (30.4–40.0) | 47.8 (44.5–51.1) | 70.1 (61.3–77.6) |

Wilson 95% confidence intervals for Figure 7.

| **Skill** | **School % (95% CI)** | **District % (95% CI)** | **Region % (95% CI)** | **State % (95% CI)** |
| --- | --- | --- | --- | --- |
| Literature review | 17.5 (15.0–20.2) | 15.9 (11.7–21.1) | 22.0 (18.3–26.2) | 33.3 (21.4–47.9) |
| Research notebook | 16.7 (14.3–19.4) | 48.1 (41.7–54.5) | 57.2 (52.5–61.8) | 60.0 (45.5–73.0) |
| Software | 39.8 (36.5–43.2) | 54.9 (48.5–61.2) | 65.7 (61.1–70.1) | 77.8 (63.7–87.5) |
| PowerPoint | 35.5 (32.3–38.9) | 29.6 (24.1–35.8) | 37.8 (33.3–42.5) | 46.7 (32.9–60.9) |
| Poster board | 62.5 (59.1–65.8) | 81.1 (75.6–85.6) | 82.7 (78.9–86.0) | 86.7 (73.8–93.7) |
| Written report | 52.7 (49.3–56.1) | 62.2 (55.9–68.2) | 58.2 (53.4–62.8) | 60.0 (45.5–73.0) |
| Interview with judges | 33.8 (30.7–37.1) | 62.2 (55.9–68.2) | 70.2 (65.7–74.4) | 75.6 (61.3–85.8) |
