# Supplementary material for: High school science fair: Characterizing student communication and presentation practices and their association with SEF outcomes
Source: PLoS One. 2026 Sep 11;21(9):e0357589. doi: 10.1371/journal.pone.0357589 (PMC13567831; doi:10.1371/journal.pone.0357589)
Supplement: Supplementary file 4 [file pone.0357589.s006.docx]

**Response to Editor and Reviewers**

**Editor**

The figures have been updated to improve clarity and we have now included statistical analyses described in the Methods section and shown in the updated figures and supporting information. Pearson chi-square tests and significance indicators have been added where comparisons involve categorical groups, and Wilson 95% confidence intervals are shown for figures in which confidence intervals provide the more appropriate description of the proportions.

**Reviewer 1**

We revised the manuscript regarding analysis of competition level to make clear that students were grouped according to the highest level of competition in which they reported competing, and that we do not know which competition levels were available to individual students. Thus, the data cannot determine whether individual students added skills as they advanced or whether students using more skills were more likely to advance.

The figures have been updated to improve clarity and we have now included statistical analyses described in the Methods section and shown in the updated figures and supporting information. Pearson chi-square tests and significance indicators have been added where comparisons involve categorical groups, and Wilson 95% confidence intervals are shown for figures in which confidence intervals provide the more appropriate description of the proportions.

**Reviewer 2**

Regarding points #1, 2, 4, 5 and 6 - The manuscript now explains how completed surveys were defined and that not every student answered every question; the figure legends identify the number of students who did not answer a particular question. We also clarify that the 4.1% shown as using zero skills represents students who completed the survey but did not select any of the listed skills. In addition, we added information that the Scienteer platform was funded by regional science fairs rather than participating schools and added the possibility that use of “PowerPoint presentation” may have missed students using Canva or other presentation software.

Regarding point #3 - We now comment in the Discussion that students already interested in STEM may be more likely to work with scientists, use research notebooks, or complete literature reviews. We also add the reviewer’s point that explicitly adding these practices to SEF scoring might encourage their use but could alternatively create an additional barrier for students who are less certain of their interest in science or have fewer educational resources. The revised manuscript emphasizes that our observational data cannot distinguish between these possibilities.

**Reviewer 3**

Regarding use of the term “skills,” we retained that terminology because it is the wording used in the survey question presented to students, but we now emphasize that the findings represent students' self-reported experiences rather than any direct evidence of proficiency in performing the skills. This distinction is particularly important because the survey asked whether students used such practices as research notebooks, PowerPoint presentations, and interviews with judges without any way to assess how proficiently students performed them.

**Journal Requirements**

Regarding the journal requirements, citation of the grant information provided is required by UT Southwestern for use of the REDCap survey and data management tool. The Robert McLemore Professorship acknowledged in the manuscript is an endowed professorship and does not have a grant number.

Also, in response to the concern about potentially identifying information in the S1 Data Set, we removed the survey timestamps and open-ended text responses. The revised S1 Data Set contains the de-identified survey responses underlying the analyses reported in the manuscript.
