# Supplementary material for: High school science fair: Characterizing student communication and presentation practices and their association with SEF outcomes
Source: PLoS One. 2026 Sep 11;21(9):e0357589. doi: 10.1371/journal.pone.0357589 (PMC13567831; doi:10.1371/journal.pone.0357589)
Supplement: Peer review history

# Peer review: Decision Letter 0

**DOI:** 10.1371/journal.pone.0357589.r001

**Authors:** Virginia Davis

3 Aug 2026

Dear Dr. Grinnell,

Please submit your revised manuscript by Sep 02 2026 11:59PM. If you will need more time than this to complete your revisions, please reply to this message or contact the journal office at plosone@plos.org. When you're ready to submit your revision, log on to https://www.editorialmanager.com/pone/ and select the 'Submissions Needing Revision' folder to locate your manuscript file.

- A letter that responds to each point raised by the academic editor and reviewer(s). You should upload this letter as a separate file labeled 'Response to Reviewers'.
- A marked-up copy of your manuscript that highlights changes made to the original version. You should upload this as a separate file labeled 'Revised Manuscript with Track Changes'.
- An unmarked version of your revised paper without tracked changes. You should upload this as a separate file labeled 'Manuscript'.

If you would like to make changes to your financial disclosure, please include your updated statement in your cover letter. Guidelines for resubmitting your figure files are available below the reviewer comments at the end of this letter.

If applicable, we recommend that you deposit your laboratory protocols in protocols.io to enhance the reproducibility of your results. Protocols.io assigns your protocol its own identifier (DOI) so that it can be cited independently in the future. For instructions see: https://journals.plos.org/plosone/s/submission-guidelines#loc-laboratory-protocols. Additionally, PLOS ONE offers an option for publishing peer-reviewed Lab Protocol articles, which describe protocols hosted on protocols.io. Read more information on sharing protocols at https://plos.org/protocols?utm_medium=editorial-email&utm_source=authorletters&utm_campaign=protocols.

As the corresponding author, your ORCID iD is verified in the submission system and will appear in the published article. PLOS supports the use of ORCID, and we encourage all coauthors to register for an ORCID iD and use it as well. Please encourage your coauthors to verify their ORCID iD within the submission system before final acceptance, as unverified ORCID iDs will not appear in the published article. *Only*  the individual author can complete the verification step; PLOS staff *cannot*  verify ORCID iDs on behalf of authors.

We look forward to receiving your revised manuscript.

Kind regards,

Virginia A. Davis, PhD

Academic Editor

PLOS One

Journal Requirements:

When submitting your revision, we need you to address these additional requirements.

1. Please ensure that your manuscript meets PLOS ONE's style requirements, including those for file naming. The PLOS ONE style templates can be found at

https://journals.plos.org/plosone/s/file?id=wjVg/PLOSOne_formatting_sample_main_body.pdf and

https://journals.plos.org/plosone/s/file?id=ba62/PLOSOne_formatting_sample_title_authors_affiliations.pdf

2. We note that the grant information you provided in the ‘Funding Information’ and ‘Financial Disclosure’ sections do not match.

When you resubmit, please ensure that you provide the correct grant numbers for the awards you received for your study in the ‘Funding Information’ section.

3. We note that there is identifying data in the Supporting Information file < S1 Data Set.xlsx>. Due to the inclusion of these potentially identifying data, we have removed this file from your file inventory. Prior to sharing human research participant data, authors should consult with an ethics committee to ensure data are shared in accordance with participant consent and all applicable local laws.

Data sharing should never compromise participant privacy. It is therefore not appropriate to publicly share personally identifiable data on human research participants. The following are examples of data that should not be shared:

-Name, initials, physical address

-Ages more specific than whole numbers

-Internet protocol (IP) address

-Specific dates (birth dates, death dates, examination dates, etc.)

-Contact information such as phone number or email address

-Location data

-ID numbers that seem specific (long numbers, include initials, titled “Hospital ID”) rather than random (small numbers in numerical order)

Data that are not directly identifying may also be inappropriate to share, as in combination they can become identifying. For example, data collected from a small group of participants, vulnerable populations, or private groups should not be shared if they involve indirect identifiers (such as sex, ethnicity, location, etc.) that may risk the identification of study participants.

Additional guidance on preparing raw data for publication can be found in our Data Policy (https://journals.plos.org/plosone/s/data-availability#loc-human-research-participant-data-and-other-sensitive-data) and in the following article: http://www.bmj.com/content/340/bmj.c181.long.

Please remove or anonymize all personal information (specific dates), ensure that the data shared are in accordance with participant consent, and re-upload a fully anonymized data set. Please note that spreadsheet columns with personal information must be removed and not hidden as all hidden columns will appear in the published file.

4. Thank you for stating the following in the Acknowledgments Section of your manuscript: [FG was supported by the UTSW Robert McLemore Professorship. Use of the REDCap survey and data management tool was facilitated by the UTSW Department of Information Resources and Clinical and Translational Science Training Program, NIH grant UL1TR001105. The REDCap funders had no role in study design, data collection and analysis, decisions to publish, or preparation of the manuscript. We are grateful to Russell Cowen and Rocky Slavin who were managers of Scienteer Technologies at the time we carried out our studies and incorporated the parental consent and SEF survey REDCap links into the Scienteer website.]

We note that you have provided funding information that is not currently declared in your Funding Statement. However, funding information should not appear in the Acknowledgments section or other areas of your manuscript. We will only publish funding information present in the Funding Statement section of the online submission form.

Please remove any funding-related text from the manuscript and let us know how you would like to update your Funding Statement. Currently, your Funding Statement reads as follows: [Use of the REDCap survey and data management tool was facilitated by the UTSW Department of Information Resources and Clinical and Translational Science Training Program, NIH grant UL1TR001105. The REDCap funders had no role in study design, data collection and analysis, decisions to publish, or preparation of the manuscript.]

Please include your amended statements within your cover letter; we will change the online submission form on your behalf.

5. If the reviewer comments include a recommendation to cite specific previously published works, please review and evaluate these publications to determine whether they are relevant and should be cited. There is no requirement to cite these works unless the editor has indicated otherwise.

6. Please review your reference list to ensure that it is complete and correct. If you have cited papers that have been retracted, please include the rationale for doing so in the manuscript text, or remove these references and replace them with relevant current references. Any changes to the reference list should be mentioned in the rebuttal letter that accompanies your revised manuscript. If you need to cite a retracted article, indicate the article’s retracted status in the References list and also include a citation and full reference for the retraction notice.

Reviewers' comments:

Reviewer's Responses to Questions

**Comments to the Author**

1. Is the manuscript technically sound, and do the data support the conclusions?

Reviewer #1: Yes

Reviewer #2: Yes

Reviewer #3: Yes

**********

2. Has the statistical analysis been performed appropriately and rigorously?

Reviewer #1: I Don't Know

Reviewer #2: I Don't Know

Reviewer #3: Yes

**********

3. Have the authors made all data underlying the findings in their manuscript fully available?

The PLOS Data policy

Reviewer #1: Yes

Reviewer #2: Yes

Reviewer #3: Yes

**********

4. Is the manuscript presented in an intelligible fashion and written in standard English?

Reviewer #1: Yes

Reviewer #2: Yes

Reviewer #3: Yes

**********

Reviewer #1: REVIEW OF PLOS One MANUSCRIPT: PONE-D-26-29968

High school science fair: Characterizing student communication and presentation practices and their association with SEF outcomes

Synopsis

This manuscript summarizes a survey-based analysis of the communication and presentation methods used by SEF participants.

Opinion

This manuscript is well-put together and the results are interesting, I have a few questions and suggested additions to the results and discussion section, but think this work is well suited for publication.

Primary criteria:

1. Does this manuscript offer original data and innovative insights?

Yes, the data is original and the insights are well-grounded, the insights aren’t hugely innovative, but are well worth publication.

2. Does the subject matter manuscript have wide scientific interest and potential applicability?

The subject matter is of interest to the general scientific audience and provides specifically useful insights for application and understanding of communication skills used by SEF participants.

3. Have the authors remembered to cite previous seminal papers on the subject?

The number of citations is acceptable, and the discussion of those results is solid, there is a significant amount of self-citation, but not to a troubling level, and the citations are appropriate, in my opinion.

4. Is the Supplementary Material (Supporting Information) helpful, appropriate and error-free?

Yes

Recommendation

Accept after minor revisions

Specific Points, Comments, and Questions

• More detail would be helpful for understanding the categories used to define the levels of competition

o Did all participants have the opportunity to participate in all levels of competition? Was there any tracking of which levels the participants were able to compete in?

• The average number of skills used by participants at different levels is a little ambiguous, is this the number of skills used by a participant AT that specific level? Or the average of all participants that got to that level?

o Was there any trends in the number of skills used by individual students AT the different levels? Did students that advanced add additional skills, or were the students that used more skills more likely to advance to the higher levels of SEF?

• It would be helpful to add error bars to the bar charts in the figures to indicate the level of statistical overlap between the groups

• Indicators for whether the differences are statistically significant between the groups would also be very helpful for interpreting the presented results

• The results presented in Figure 4, seem to indicate that there are potentially significant differences in the total number of skills used across demographic groups in addition to the changes in which skills are used, a Figure 2-style plot with the different demographics would be interesting to see, or at least a note about whether the differences are statistically significant

Reviewer #2: This article shows an interesting correlation between interest in STEM and particular techniques/skills used by students while completing science fair projects, which a strong association with lab notebooks in particular and is worthy of being published in PLOS one. Data collected seems to have been treated appropriately, and the limitations are listed in the manuscript. My suggestions for revisions are below.

1. In figures 3, 4, 8, and 9 there is a note that a certain number of students didn't answer "this" question. I think it is unclear upon first reading if you mean the question being measured (about techniques used) or the demographic question etc. After this showing up in multiple figures I was able to figure out you meant they didn't answer a second question, but I think there might be better verbiage to explain which question isn't answered.

2. In figure 2, it is shown that 4.1% of students said they didn't use any of the skills listed. Were these surveys just entirely blank, or did they just skip the question? It seems unlikely that a science fair project could be completed without any of the techniques listed. This gave me pause that perhaps they were affecting your percentages, when the students didn't answer the survey.

3. The major issue I see is the suggestion that inclusion of notebooks and literature reviews into scoring rubrics would cause students to give those techniques more attention (end of third discussion paragraph), with the implication that outcomes like excitement in STEM and interest in STEM careers. I feel this statement implies that there is causation rather than just association with those techniques and interest. It is not mentioned in the manuscript that there is a possibility that students who already are interested in STEM may be more likely to engage with a scientist, use lab notebooks, or complete a literature review. I understand the suggestion, as since there is no way to prove causality with these data, as mentioned clearly by the authors, it stands to reason that attempting to include these in rubrics would help to elucidate that relationship. My worry would be that students, teachers, and potentially parents, would be less likely to participate if there is a higher barrier to participate at a fundamental level. Students who are interested already complete those tasks, and students who may be less sure of their interest might be discouraged by the additional layer of participation if there is no causal link present. I just think this suggestion implies causality. As a scientist, I agree with this statement but without any clear evidence other than my opinion and the association you've shown. If this statement stays included, which I understand, I think a more thorough discussion of the limitation of being unable to separate students with interest before the fair and potential downside of including those is warranted.

4. For the limitations discussion, is Scienteer a paid service? I am unsure of the pay structure there, but if schools have to pay to use it then I would say that is another level of limitation for your student population as many schools may skip that expense.

5. The choice of "PowerPoint presentation" as one option rather than a "Slide Show" or "presentation" may be a limitation as well. Many students no longer use PowerPoint and use Canva or some other software. You may be missing students who are indeed making a computer generated slide show/slide deck, but don't realize that is what you are asking by putting "PowerPoint".

Overall, I think this manuscript shows associations that are worth sharing with the greater community and may help teachers to better curate their "best practices" and is worthy of publication in PLOS One.

Reviewer #3: The manuscript investigates students’ communication experiences in the context of Science and Engineering Fairs (SEFs). The authors draw on students’ responses to a voluntary post-fair survey about the communication practices and presentation formats they engaged with while developing and presenting their projects. They then examine associations between these responses and several factors, including students’ demographic characteristics, external sources of support, and perceived benefits of participating in SEFs.

One particularly noteworthy finding is that core SEF requirements (namely, preparing a poster board and participating in interviews with judges) are closely associated with the forms of communication students reported using. Because these formats are required components of participation, their prevalence may reflect the structure of the fairs rather than students’ independent selection of communication media. Nevertheless, this finding creates an important opportunity to consider how the design and requirements of SEFs might encourage engagement with a broader range of communication practices.

Methodologically, the manuscript provides sufficient detail about participant eligibility and recruitment. The authors explain that students completed the post-fair survey voluntarily, without pressure or incentives, and transparently describe the study procedures and how consent was obtained from students and their parents. They also appropriately acknowledge the limitations of generalizing from this sample to the broader population of students participating in SEFs in the United States.

The analyses are straightforward, and the authors report statistical significance when differences between groups are identified. The manuscript is also generally careful not to make causal claims or extend its conclusions beyond what can reasonably be inferred from the data.

One point that would benefit from further clarification is the use of the term “skill” in the survey and throughout the manuscript. Some of the corresponding response options—such as research notebooks, PowerPoint presentations, and interviews with judges—appear to refer to artifacts, presentation formats, or activities rather than skills themselves. Skills or practices might instead include documenting a research process, designing a visual presentation, or orally explaining and defending a project.

Relatedly, the survey appears to capture students’ self-reported participation in particular communication activities rather than directly assessing their communication proficiency. The manuscript would therefore benefit from consistently distinguishing among exposure to a communication format, engagement in a communication practice, perceived skill development, and demonstrated communication competence. The authors already acknowledge that students may have interpreted some survey options differently from what was intended, and I do not believe that this issue substantially limits the paper’s overall findings or contribution. Nevertheless, greater conceptual precision would strengthen both the presentation and interpretation of the results.

Overall, I find the manuscript methodologically sound and its conclusions appropriately supported by the evidence. My main recommendation is that the authors clarify the constructs measured by the survey, particularly the distinction among communication skills, practices, formats, and artifacts, and ensure that the findings are consistently framed as students’ self-reported experiences rather than direct evidence of demonstrated communication skills.

**********

what does this mean?). If published, this will include your full peer review and any attached files.

If you choose “no”, your identity will remain anonymous but your review may still be made public.

**Do you want your identity to be public for this peer review?**  For information about this choice, including consent withdrawal, please see our Privacy Policy

Reviewer #1: No

Reviewer #2: No

Reviewer #3: No

**********

[NOTE: If reviewer comments were submitted as an attachment file, they will be attached to this email and accessible via the submission site. Please log into your account, locate the manuscript record, and check for the action link "View Attachments". If this link does not appear, there are no attachment files.]

To ensure your figures meet our technical requirements, please review our figure guidelines: https://journals.plos.org/plosone/s/figures

You may also use PLOS’s free figure tool, NAAS, to help you prepare publication quality figures: https://journals.plos.org/plosone/s/figures#loc-tools-for-figure-preparation.

NAAS will assess whether your figures meet our technical requirements by comparing each figure against our figure specifications.

# Author comment: Author response to Decision Letter 1

**DOI:** 10.1371/journal.pone.0357589.r002

18 Aug 2026

Editor

The figures have been updated to improve clarity and we have now included statistical analyses described in the Methods section and shown in the updated figures and supporting information. Pearson chi-square tests and significance indicators have been added where comparisons involve categorical groups, and Wilson 95% confidence intervals are shown for figures in which confidence intervals provide the more appropriate description of the proportions.

Reviewer 1

We revised the manuscript regarding analysis of competition level to make clear that students were grouped according to the highest level of competition in which they reported competing, and that we do not know which competition levels were available to individual students. Thus, the data cannot determine whether individual students added skills as they advanced or whether students using more skills were more likely to advance.

The figures have been updated to improve clarity and we have now included statistical analyses described in the Methods section and shown in the updated figures and supporting information. Pearson chi-square tests and significance indicators have been added where comparisons involve categorical groups, and Wilson 95% confidence intervals are shown for figures in which confidence intervals provide the more appropriate description of the proportions.

Reviewer 2

Regarding points #1, 2, 4, 5 and 6 - The manuscript now explains how completed surveys were defined and that not every student answered every question; the figure legends identify the number of students who did not answer a particular question. We also clarify that the 4.1% shown as using zero skills represents students who completed the survey but did not select any of the listed skills. In addition, we added information that the Scienteer platform was funded by regional science fairs rather than participating schools and added the possibility that use of “PowerPoint presentation” may have missed students using Canva or other presentation software.

Regarding point #3 - We now comment in the Discussion that students already interested in STEM may be more likely to work with scientists, use research notebooks, or complete literature reviews. We also add the reviewer’s point that explicitly adding these practices to SEF scoring might encourage their use but could alternatively create an additional barrier for students who are less certain of their interest in science or have fewer educational resources. The revised manuscript emphasizes that our observational data cannot distinguish between these possibilities.

Reviewer 3

Regarding use of the term “skills,” we retained that terminology because it is the wording used in the survey question presented to students, but we now emphasize that the findings represent students' self-reported experiences rather than any direct evidence of proficiency in performing the skills. This distinction is particularly important because the survey asked whether students used such practices as research notebooks, PowerPoint presentations, and interviews with judges without any way to assess how proficiently students performed them.

Journal Requirements

Regarding the journal requirements, citation of the grant information provided is required by UT Southwestern for use of the REDCap survey and data management tool. The Robert McLemore Professorship acknowledged in the manuscript is an endowed professorship and does not have a grant number.

Also, in response to the concern about potentially identifying information in the S1 Data Set, we removed the survey timestamps and open-ended text responses. The revised S1 Data Set contains the de-identified survey responses underlying the analyses reported in the manuscript.

Attachment Submitted filename: Response to Reviewers.docx

# Editor report: Decision Letter 1

**DOI:** 10.1371/journal.pone.0357589.r003

**Authors:** Virginia Davis

19 Aug 2026

High school science fair: Characterizing student communication and presentation practices and their association with SEF outcomes

PONE-D-26-29968R1

Dear Dr. Grinnell,

We’re pleased to inform you that your manuscript has been judged scientifically suitable for publication and will be formally accepted for publication once it meets all outstanding technical requirements.

Within one week, you’ll receive an e-mail detailing the required amendments. When these have been addressed, you’ll receive a formal acceptance letter and your manuscript will be scheduled for publication.

An invoice will be generated when your article is formally accepted. Please note, if your institution has a publishing partnership with PLOS and your article meets the relevant criteria, all or part of your publication costs will be covered. Please make sure your user information is up-to-date by logging into Editorial Manager at Editorial Manager® and clicking the ‘Update My Information' link at the top of the page. For questions related to billing, please contact billing support.

If your institution or institutions have a press office, please notify them about your upcoming paper to help maximize its impact. If they’ll be preparing press materials, please inform our press team as soon as possible -- no later than 48 hours after receiving the formal acceptance. Your manuscript will remain under strict press embargo until 2 pm Eastern Time on the date of publication. For more information, please contact onepress@plos.org.

Kind regards,

Virginia A. Davis, PhD

Academic Editor

PLOS One

Additional Editor Comments (optional):

Reviewers' comments:

# Editor report: Acceptance letter

**DOI:** 10.1371/journal.pone.0357589.r004

**Authors:** Virginia Davis

PONE-D-26-29968R1

PLOS One

Dear Dr. Grinnell,

I'm pleased to inform you that your manuscript has been deemed suitable for publication in PLOS One. Congratulations! Your manuscript is now being handed over to our production team.

At this stage, our production department will prepare your paper for publication. This includes ensuring the following:

* All references, tables, and figures are properly cited

* All relevant supporting information is included in the manuscript submission,

* There are no issues that prevent the paper from being properly typeset

You will receive further instructions from the production team, including instructions on how to review your proof when it is ready. Please keep in mind that we are working through a large volume of accepted articles, so please give us a few days to review your paper and let you know the next and final steps.

Lastly, if your institution or institutions have a press office, please let them know about your upcoming paper now to help maximize its impact. If they'll be preparing press materials, please inform our press team within the next 48 hours. Your manuscript will remain under strict press embargo until 2 pm Eastern Time on the date of publication. For more information, please contact onepress@plos.org.

You will receive an invoice from PLOS for your publication fee after your manuscript has reached the completed accept phase. If you receive an email requesting payment before acceptance or for any other service, this may be a phishing scheme. Learn how to identify phishing emails and protect your accounts at https://explore.plos.org/phishing.

If we can help with anything else, please email us at customercare@plos.org.

Thank you for submitting your work to PLOS One and supporting open access.

Kind regards,

PLOS One Editorial Office Staff

on behalf of

Dr. Virginia A. Davis

Academic Editor

PLOS One
